# 

**Published:** 1917-11-03

**Authors:** 


RATES OF SUBSCRIPTION: Twelve months, 6/6; Six months, 4/-; three months, 2/-, post free to any address in the United Kingdom. Abroad, Twelve months, 8/8; Six months, 5/-; Three months, 2/6, post free. **THE HOSPITAL "Index" to Volume LXI., October 1916 to March 1917, is now ready, price 2½d. per copy, post free.** Address The Manager, THE HOSPITAL, "The Hospital" Building, 28/29 Southampton Street, Strand, London, W.C. 2.

**Publications.**

### E. & S. LIVINGSTONE, MEDICAL PUBLISHERS, 17 TEVIOT PLACE, EDINBURGH.

Fifth Edition, Revised and Enlarged, with new Illustrations. Crown 8vo, Cloth, pp. 576. 10s. net. Postage 5d.
**WHEELER'S HANDBOOK OF MEDICINE.** By W. R. JACK, B.Sc., M.D., F.R.C.P.S.G.

Crown 8vo, Cloth, pp. 403+16. 80 Illustrations, price 6s. net.
**MANUAL OF HYGIENE. For Students and Nurses.** By JOHN GLAISTER, M.D., D.P.H. (Camb.), Professor of Public Health, University of Glasgow.

Cloth, 16mo. 1s. 6d. net. Postage 2d.
**THE NURSES' GUIDE TO PRESCRIPTION READING.** Designed for the Use of Nurses. By J. G. H.

Crown 8vo, pp. 295. Illustrated by Radiograms. 4s. 6d. net.
**CLINICAL LECTURES IN MEDICINE. For Nurses and Junior Practitioners.** By D. CHALMERS WATSON, M.D., F.R.C.P., &c.

**CATECHISM SERIES.** 1s. 3d. per Part. 50 Parts in all. These include the following:—Anatomy, Bacteriology, Botany, Chemistry, Gynecology, Histology, Materia Medica, Medicine, Midwifery, Operative Surgery, Pathology, Physics, Physiology, Public Health, Surgery, Surgical Anatomy and Operations, Toxicology, and Zoology.
Medicine, Pathology, Public Health, and Surgery are bound in one volume. Cloth. Price 5s. 3d. net.

Crown 8vo, Cloth, pp. 110. Illustrations. 2s. net.
**MODERN WOUND TREATMENT AND CONDUCT OF AN OPERATION.** By Sir GEORGE T. BEATSON, K.C.B., B.A.Camb., M.D.Edin.

Fifth Edition. With numerous Illustrations. 2s. net. Postage 3d.
**THE EXAMINATION OF THE URINE AND OTHER CLINICAL SIDE ROOM METHODS.** By ANDREW FERGUS HEWAT, M.B., Ch.B., M.R.C.P.Edin.

Fifth Edition, Revised according to the New *B.P.* 2s. net. Postage 2d.
**T E STUDENT'S POCKET PRESCRIBER AND GUIDE TO PRESCRIPTION WRITING.** By D. M. MACDONALD, M.D.

Cr. 8vo, Cloth. 2 vols. pp. 1064. 266 Illustrations. 10s. net. Postage 7d.
**APPLIED ANATOMY: Surgical, Medical, and Operative.** By A. A. SCOT SKIRVING, C.M.G., F.R.C.S.Edin.

Fourth Edition, Revised according to the New *B.P.* 1s. 3d. net. Postage 1d.

**POSOLOGICAL TABLES.** By WILLIAM CRAIG, M.D.

Second Edition, Crown 8vo, Cloth, pp. 350. Enlarged, with new Illustrations, Coloured and Plain. 7s. 6d. net. Postage 5d.
**A MANUAL OF SURGICAL ANATOMY.** By Capt. C. R. WHITTAKER, R.A.M.C., F.R.C.S.Edin., F.R.S.E.

Crown 8vo, 158 pp. With 38 Illustrations. Paper 1s. 6d. Cloth 2s. net.
**THE CARE OF INFANTS AND YOUNG CHILDREN.** By A. DINGWALL FORDYCE, M.D., F.R.C.P.E., Extra Physician, Royal Hospital for Sick Children, Edinburgh.

Fourth Edition, Crown 8vo, Cloth, pp. 288. With many Coloured Illustrations. 7s. net. Postage 5d.
**ANATOMY OF THE BRAIN AND SPINAL CORD.** By J. RYLAND WHITAKER, B.A., M.B.Lond., F.R.C.P.Edin.

**CATECHISM SERIES ON ALL MEDICAL, SURGICAL, AND ALLIED SUBJECTS.** 1s. 3d. net per Part. 50 Parts in all.

Crown 8vo, Cloth, pp. 182. 2s. 6d. net. Postage 4d.
**BACK INJURIES, and their Significance under the Workmen's Compensation and other Acts.** By ARCHIBALD M'KENDRICK, F.R.C.S.E., &c., Surgeon-in-Charge of Surgical X-Ray Department, Royal Infirmary, Edinburgh.

**CATALOGUES SENT POST-FREE ON APPLICATION.**

By SIR WILLIAM BENNETT, K.C.V.O., F.R.C.S.

***Crown 8vo. 5s. net.***

### INJURIES AND DISEASES OF THE KNEE JOINT

With 34 Illustrations.

NISBET & CO.

***Also, 8vo. 6s.***

FOURTH EDITION.

**Massage and Movements in Recent Fractures; Sprains and their Consequences; Rigidity of the Spine and the Management of Stiff Joints.**

With 23 Illustrations.

LONGMANS, GREEN & CO.

viii + 320 pages. Price **6s.** net.

**THE TREATMENT OF DISEASES OF THE SKIN.**
By W. KNOWSLEY SIBLEY, M.A., M.D., B.C. (Camb.), M.R.C.P., Physician to St. John's Hospital for Diseases of the Skin, London. Illustrated with 16 Full-page Plates and 14 Figures.
LONDON: EDWARD ARNOLD, 41 & 43 MADDOX STREET, W.

*Large 8vo. Profusely Illustrated.* 8/6 *net, or Interleaved for Notes,* 9/6 *net.*
**INTRODUCTION TO SURGERY.**
By PROF. RUTHERFORD MORISON, M.A., M.B., F.R.C.S. Edin. and Eng. *Prof. of Surg. Univ. of Durham Coll. of Med., Newcastle-on-Tyne.*
With 146 Original Illustrations in the Text, together with 5 Coloured Plates.
"Extremely suggestive . . . very much that is not to be found in most works. . . . Singularly successful . . . has a reason and character of its own."—*Lancet.*
"Can be read from end to end, not only with pleasure, but with profit, by the well-educated practitioner."—*South African Med. Record.*

FOR THE PROFESSIONAL NURSE.

*THIRD EDITION.* *Price 3s. 9d. post free.*

**SICKROOM COOKERY AND HOSPITAL DIET,**
with Special Recipes for Convalescent & Diabetic Patients.
By MAUDE EARLE.
With NOTES ON THE FEEDING OF INFANTS.
LONDON: SPOTTISWOODE, BALLANTYNE & CO. LTD., 1 New-street Square, E.C. 4.

Now Ready. Fourth Edition, Revised and Enlarged. Price **4s. 6d.** net.
**INSANITY IN EVERYDAY PRACTICE.**
By E. G. YOUNGER, M.D., M.R.C.P., D.P.H.
London: BAILLIÈRE, TINDALL & COX, 8 Henrietta Street, Covent Garden.

